# Altered Maternal Serum Matrix Metalloproteinases MMP-2, MMP-3, MMP-9, and MMP-13 in Severe Early- and Late-Onset Preeclampsia

**DOI:** 10.1155/2017/6432426

**Published:** 2017-07-17

**Authors:** Marzena Laskowska

**Affiliations:** Chair and Department of Obstetrics and Perinatology, Medical University of Lublin, Jaczewskiego 8, 20-950 Lublin, Poland

## Abstract

**Objective:**

The aim of this study was to determine whether maternal serum matrix metalloproteinases 2, 3, 9, and 13 levels differ in early- and late-onset preeclampsia and uncomplicated pregnancies.

**Patients and Methods:**

The study was carried out in 125 pregnant women (29 with early-onset preeclampsia; 31 preeclamptic patients with late-onset preeclampsia; and 65 healthy pregnant controls). Levels of MMP-2, MMP-3, MMP-9, and MMP-13 were measured in the maternal serum using an enzyme-linked immunosorbent assay.

**Results:**

Maternal serum MMP-2 levels in both the groups of preeclamptic women were significantly higher than those in the controls. Levels of MMP-3 were significantly higher in preeclamptic patients with early-onset disease; however, the MMP-3 levels in patients with late-onset preeclampsia were similar to those observed in the control subjects. MMP-9 levels were lower whereas the levels of MMP-13 were higher in both preeclamptic groups of pregnant women than in the healthy controls, but these differences were statistically insignificant.

**Conclusions:**

One important finding of the present study was that MMP-3 appears to be involved solely in early-onset preeclampsia, but not in late-onset preeclampsia. Higher levels of MMP-2 and MMP-13 and lower levels of MMP-9 seem to be related to both early- and late-onset severe preeclampsia.

## 1. Introduction

Although there have been extensive studies of preeclampsia, its aetiology remains unclear [1–6]. However, it is widely accepted that shallow placentation and impaired spiral arteries precede an increase in secretion of antiangiogenic factors and endothelial cells dysfunction, which are underlying mechanisms of preeclampsia [2–4, 7, 8]. The conception of the key role of the placenta in preeclampsia is strongly supported by the fact that currently known and the solely causal treatment of this specific severe pregnancy disorder is the delivery of a newborn and placenta [3, 4].

Reister et al. [3] presented that insufficient invasion of extravillous trophoblast in the spiral artery wall plays an important role in the development of early-onset preeclampsia with IUGR. However, recent studies by Redman et al. [9] show that abnormal placental perfusion and oxidative syncytiotrophoblast stress may contribute to both early and late preeclampsia with special importance of poor placentation in the early-onset disease and greater significance of the microvillus overcrowding and functional limits of placental growth as a cause of late-onset preeclampsia [3, 4, 9].

Metalloproteinases zinc-dependent enzymes have been reported to be involved in extracellular matrix remodelling and processes of placental angiogenesis with structural spiral arteries transformation, which precedes proper trophoblast invasion [2, 5, 6, 10]. Regulation of angiogenesis, a multistage process including activation and proliferation of vascular endothelial cells and degradation of extracellular matrix, which allows the invasion of proliferating cells into surrounding tissues is one of the most important functions of metalloproteinases [2, 3, 5].

The increase of both the local expression and activity of some metalloproteinases was observed in pathologically changed human arteries and atherosclerotic plaques. Two gelatinases: MMP-2 and MMP-9, stromelisyn-1 (MMP-3), and collagenase MMP-13 belong to a group of metalloproteinases directly linked to the process of blood vessels remodelling [11]. The key importance of MMP-2 in development of hypertension is being discussed due to its capability of converting edothelin into its active form in vascular smooth muscle cells, as well as its participation in degradation of adrenomedullin, which is connected with process of vasodilatation, especially important in preeclampsia [11–14]. MMP-9 plays a role in metabolism of basement membrane of blood vessels and is activated in the wall of blood vessels. MMP-3 is a considerable risk factor of vascular disorder and incident coronary heart disease. Moreover, MMP-3 participates in the process of trophoblast invasion in healthy pregnancies [11–14].

The results of previous research into the role and expression pattern of metalloproteinases in pregnancies complicated by preeclampsia are not unambiguous.

**Figure 1:**
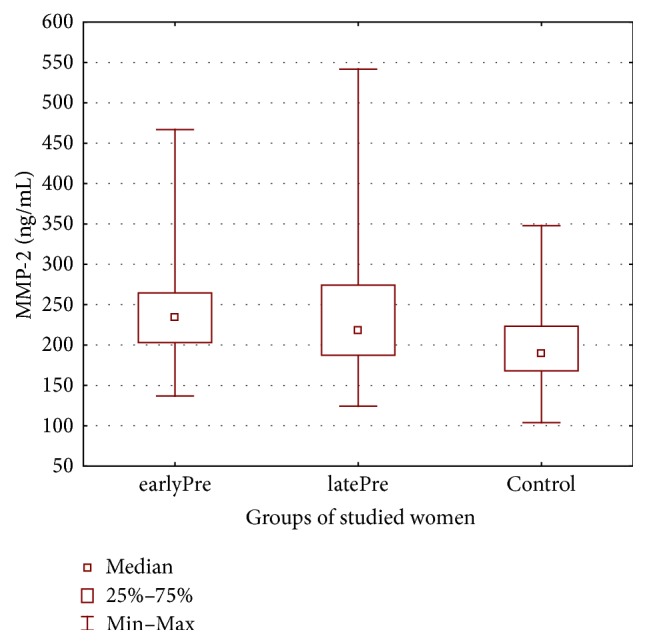
Maternal serum matrix metalloproteinases-2 in both preeclamptic groups of women and in healthy controls.

**Figure 2:**
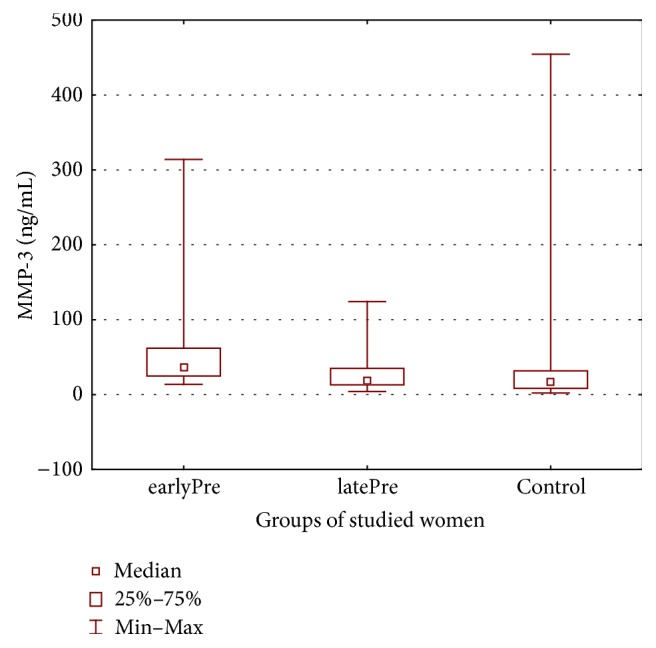
Maternal serum matrix metalloproteinases-3 in both preeclamptic groups of women and in healthy controls.

**Figure 3:**
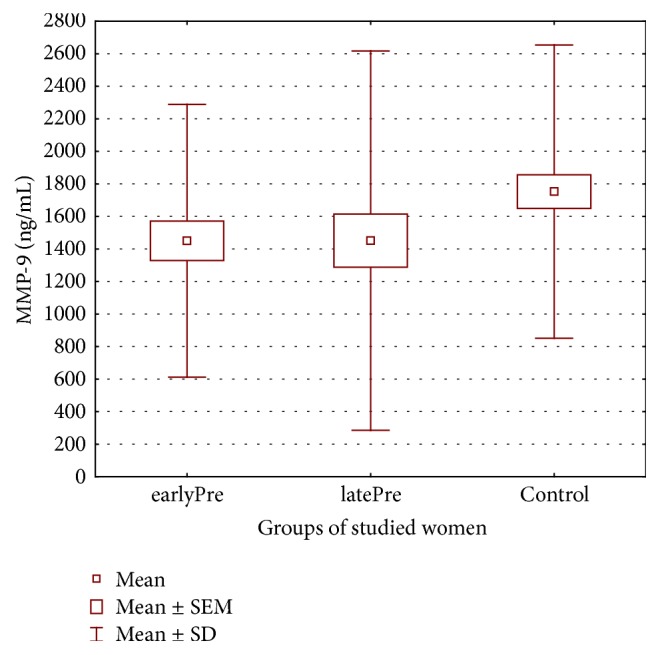
Maternal serum matrix metalloproteinases-9 in both preeclamptic groups of women and in healthy controls.

**Figure 4:**
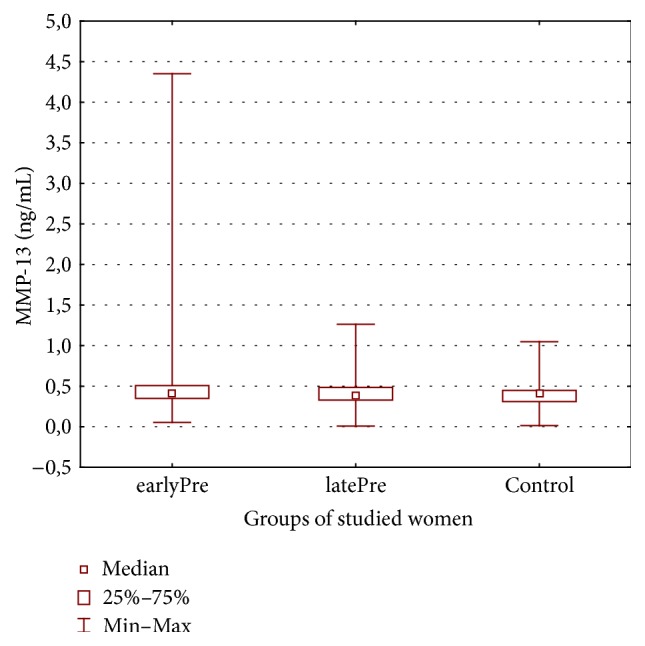
Maternal serum matrix metalloproteinases-13 in both preeclamptic groups of women and in healthy controls.

Therefore, the aim of this study was to explore whether severe preeclampsia is associated with changed maternal serum matrix metalloproteinases MMP-2, MMP-3, MMP-9, and MMP-13 levels (see Figures 1, 2, 3, and 4) and whether there are differences in these metalloproteinases levels in women with early- and late-onset preeclampsia.

## 2. Materials and Methods

### 2.1. Patients

The patients who had been referred to the tertiary-level academic unit for further treatment and perinatal surveillance because of preeclampsia without any signs of labour were offered participation in this study. Informed consent was obtained from each woman for the participation in the study and for peripheral blood sampling and the protocol for the study was approved by the Bioethical Board of the Medical University of Lublin* (KE-0254/51/2010)*.

A total of 60 pregnant women at 26–38 weeks of gestation, with pregnancies complicated by severe preeclampsia (29 women with early-onset preeclampsia and 31 women with late-onset preeclampsia) and 65 pregnant women with normotensive, uncomplicated third-trimester pregnancies were included in the study. Blood pressure had been measured on each pregnant patient's arm supported and positioned at the level of her heart on two occasions at least 4 hours apart, with using the first Korotkoff sound for evaluation of the systolic blood pressure and the fifth for assessment of the diastolic blood pressure [1].

Severe hypertension was defined as systolic blood pressure ≥ 160 mmHg and diastolic blood pressure ≥ 110 mmHg.

Severe preeclampsia was defined according to new American College of Obstetricians and Gynecologists' guidelines as new-onset hypertension in the second half of pregnancy accompanied by new-onset proteinuria and/or haematologic abnormalities (thrombocytopenia, microangiopathic haemolysis), cerebral or visual disturbances, renal or liver impaired function, HELLP syndrome (haemolysis, elevated liver enzymes, low platelet count, and right-upper quadrant pain), or pulmonary oedema [15]. Early-onset preeclampsia was defined as being diagnosed before 34th weeks of gestation. Preeclampsia was classified as late-onset preeclampsia as occurring after completed 34 weeks of gestation [16, 17].

None of the preeclamptic pregnant patients had been affected by chronic hypertension, renal disorders, or proteinuria before pregnancy and all of them were normotensive before the 20th week of gestation. All preeclamptic women were normotensive 3 months after labour [9].

None of the 65 healthy controls had signs of elevated blood pressure or other pregnancy disorders or complications and all of them gave birth to healthy infants. None of the patients from this group suffered from proteinuria. All patients in the study were nonsmokers.

All women were with singleton pregnancies. All cases of premature rupture of membranes, chorioamnionitis, inflammatory conditions, and other medical diseases such as chronic hypertension, diabetes mellitus, autoimmune disorders, and heart, renal, or liver diseases prior to the pregnancy were excluded from the study.

### 2.2. Sampling

Upon admission data were collected concerning maternal anthropometric parameters and medical history was taken. In all patients, blood was drawn for assessment of serum levels of matrix metalloproteinases MMP-2, MMP-3, MMP-9, and MMP-13. Ten millilitres of blood was collected by venipuncture from each preeclamptic woman and from each woman in the control group before any drug administration. The blood samples were placed in sterile tubes at 4°C and centrifuged for 15 min at 1500 ×g. Afterwards, the serum samples were stored at –70°C until assayed.

### 2.3. Measurement Serum Maternal Metalloproteinases Levels

The levels of maternal serum MMP-2, MMP-3, MMP-9, and MMP-13 were determined using a specific and sensitive commercially available enzyme-linked immunosorbent assays (ELISA assays) according to the manufacturer's instructions (R&D Systems, Albington, UK, for MMP-2 and Bender MedSystem, Vienna, Austria, for MMP-3, MMP-9, and MMP-13).

For MMP-2 a minimum detectable level was 0.047 ng/mL with intra-assay 5.5% and interassay 6.9% precision rate. For MMP-3 a minimum detectable level was 0.3 ng/mL with intra-assay 7.3% and interassay 8.8% precision rate. For MMP-9, the lowest detectable concentration was 0.5 ng/mL and for MMP-2 the minimum detectable level was 0.047 ng/mL with intra-assay 7.3% and interassay 10.2% precision rate. For MMP-13 the minimum detectable level was 0.18 ng/mL with intra-assay 7.4% and interassay 8.2% precision.

### 2.4. Statistical Methods

In the statistical analysis, results were expressed as mean ± SD or SEM or as median values and were statistically analysed with the computer program “Statistica 8” using the Shapiro-Wilk test for the normally distributed data and equality of variance by Levene test and, subsequently, two-tailed* t-*tests, or (in unequal variance) the Cochran-Cox test.

The ANOVA and Kruskal-Wallis tests were used to test for differences among 3 independent groups. A statistically significant effect in ANOVA was followed by follow-up post hoc Tukey's test in order to assess which group is different. A *p* value of less than 0.05 was considered to be significant.

## 3. Results

In the present study 125 pregnant women at 26–38 weeks of gestation had been investigated. Sixty patients had a diagnosis of severe preeclampsia and were divided into two study groups with early- and late-onset disease (the earlyPre and the latePre groups), and sixty-five of them had healthy normotensive pregnancies without any complications (the control group).

The general demographic characteristics and obstetric history of the study population are shown in Table 1. There were no statistically significant differences in maternal age and height in patient profiles between groups. Creatinine and urea levels were normal in all the patients. Maternal weight and BMI were higher in the two groups of preeclamptic patients. Systolic and diastolic blood pressure and mean arterial blood pressure were significantly higher in the two studied groups of preeclamptic women than in the control group. Lower gestational age at birth and birth weight of infants were observed in both groups of preeclamptic patients in comparison with the control subjects.

Table 2 displays the metalloproteinase levels in maternal serum samples in both the preeclamptic groups of the studied women with early- and late-onset of preeclampsia and in the healthy control group.

In the present study it was found that both groups of preeclamptic patients with early- and late-onset preeclampsia had significantly higher MMP-2 levels than the healthy controls and were distributed as follows: 242.93  ±  68.64 ng/mL (136.88–466.90 ng/mL) in earlyPre, 234.39.7 ± 79.18 ng/mL (124.19–514.79 ng/mL) in late-onset preeclampsia, and 195.30 ± 43.53 ng/mL (103.88–347.83 ng/mL) in the control group.

One important finding of the present study was that MMP-3 levels were significantly higher in the earlyPre group than in the preeclamptic patients with late-onset preeclampsia (latePre group) and the controls. There were no differences in MMP-3 levels between the group of patients with pregnancy complicated by late-onset preeclampsia and the control subjects.

To the best of my knowledge, this is the first study that has detected high levels of MMP-3 in patients with early-onset preeclampsia and unaltered levels of MMP-3 in patients with late-onset preeclampsia.

The mean values were 63.54 ± 71.58 ng/mL (13.65–314.14 ng/mL) in the earlyPre group and 27.91 ± 24.99 ng/mL (4.16–124.17 ng/mL) in the latePre group compared with 29.31 ± 58.79 ng/mL (2.046–454.53 ng/mL) in the control group.

Maternal serum levels of MMP-9 were lower in patients with pregnancies complicated by preeclampsia. The levels of MMP-13 were higher in preeclamptic women, especially in patients with early-onset preeclampsia. But these differences were statistically insignificant. The mean values of MMP-9 and MMP-13 were 1450.12 ± 838.01 ng/mL (243.68–3383.13 ng/mL) and 0.590 ± 0.681 ng/mL (0.054–4.351 ng/mL) in the earlyPre group, 1450.86 ± 1165.93 ng/mL 325.07–5430.0 ng/mL) and 0.482 ± 0.278 (0.008–1.264 ng/mL) in the latePre group, and 1725.53 ± 901.54 ng/mL (336.83–4569.2 ng/mL) and 0.427 ± 0.228 ng/mL (0.014–1.047 ng/mL) in the healthy controls, respectively.

## 4. Discussion

The present study revealed higher levels of MMP-2 and MMP-13 in both groups of the studied patients with early- and late-onset preeclampsia in comparison with healthy normotensive controls. The levels of MMP-9 were lower in the patients with pregnancy complicated by severe preeclampsia. MMP-3 levels were significantly higher in the patients with early-onset preeclampsia than in the late-onset preeclampsia and the controls. There were no differences in MMP-3 levels between the patients with pregnancy complicated by late-onset preeclampsia and the control subjects.

Similar results of higher levels of MMP-2 and lower levels of MMP-9 in maternal serum of preeclamptic women were found by Montagnana et al. [18]. These authors noticed higher levels of metalloproteinase-2 in preeclamptic women than in healthy pregnant controls as well as in nonpregnant women. The levels of MMP-9 were higher in pregnant women than in nonpregnant controls [18]. Narumiya et al. [19] indicated elevated MMP-2 and MMP-9 levels below detection in pregnancies complicated by preeclampsia.

Palei et al. observed increased activity of MMP-9 and MMP-9/TIMP-1 ratio in pregnancies complicated by gestational hypertension, but not in those with preeclampsia whereas MMP-2 did not show any differences with respect to healthy pregnancies and pregnancies complicated by hypertension or preeclampsia [20].

However, our studies showed different results.

Although Gerlach et al. [21] do not recommend measurement of MMPs in blood serum but in plasma because of the fact that MMP-9 levels observed in serum can be higher comparing to these observed in plasma which is due to additional release of this metalloproteinase by platelets and leucocytes.

However, taking into account the lower serum MMP-9 levels in preeclamptic pregnancies obtained in our study, it can be supposed that the plasma MMP-9 levels in these women would be even lower, which further confirms our findings. For our defence we can assure that all collected samples of serum were prepared and frozen at a temperature of 70°C to an analysis directly after taking blood, in order to avoid elevating of MMP-9 levels artificially.

Reiter indicates that impaired formation of uteroplacental arteries is associated with an increased risk of preeclampsia [3]. It has been also suggested that MMP-2 may reflect endothelial dysfunction in preeclamptic pregnancies [22] and may lead to abnormal vasoactive peptides activity and an enhanced vasoconstriction [23].

However, it is difficult to propose possible clinical application of tissue inhibitors of metalloproteinases (TIMPs) in treatment, since some reports reveal that TIMP-2 are capable of blocking the vasodilator effect of MMP-2 in small blood vessels, which additionally would potentiate pathological mechanism observed in preeclampsia [24–27]. Moreover, it should be underlined that this vasodilator effect is even more pronounced in pregnancies complicated by preeclampsia in comparison to healthy pregnancies and reaction in nonpregnant women [24, 25].

At the same time, results of the present study can suggest the importance of increased MMP-2 levels as a defence mechanism against increased vasoconstriction observed in small prearteriolar vessels in pregnancy complicated by preeclampsia [26].

However, different results were presented by Palei et al., who did not observe changes in MMP-2 activity neither in pregnancies complicated by gestational hypertension nor by preeclampsia with respect to healthy pregnancies [20].

Despite initially promising signals about possible use of MMPs inhibitors, it is hard to clearly determine their role in medical treatment [24–27]. Studies carried out on animals models show that the use of MMPs inhibitors in cancer treatment did not bring expected results [25]. In addition, they reveal a possibility of adverse side-effects, though not related to inhibiting of MMP [25].

MMP-9 is known to participate in trophoblast cells invasion and to be involved in the formation of new blood vessels and thus it is called a trigger of angiogenesis [25]. In addition, the development of very small blood vessels and process of intravasation require the presence of MMP-9 [13].

Coolman et al. [28] put forward that an increase in MMP-9 levels in normal pregnancy is essential for the development of proper maternal-fetal interface. Su et al. [23] observed that decreased expression of MMP-2 and MMP-9 leads to lower ability of trophoblast invasion.

In view of the literature data, the results of the present study may suggest the importance of lower maternal serum MMP-9 levels in abnormal development of blood vessels at the interface between mother and fetus in pregnancies complicated by severe preeclampsia. Higher levels of MMP-2 seem to be responsible for the endothelial damage and severe vasoconstriction observed in severe preeclampsia. At the same time, taking into account suggestions that MMP-2 is taking part in process of vasodilatation, its elevated concentrations could constitute an attempt to compensate alterations observed in pregnancies complicated by preeclampsia.

Different results were also presented by Poon et al. [29], who observed increased MMP-9 levels in pregnancies complicated by preeclampsia. Also Lockwood et al. [30] suggest that higher expression of MMP-9 in decidual cells could play an important role in preeclampsia. On the contrary, Mckirdy and Marks [31] demonstrated negative relationship between gestational age and active MMP-9 expression throughout normal gestation, but no significant differences were reported between MMP-2 and MMP-9 levels in the placenta from healthy, IUGR, and preeclamptic pregnancies. However, Prochazka et al. [32] reported no statistically significant differences in the levels of MMP-2 and MMP-9 in preeclamptic pregnancies irrespective of the trimester when compared with healthy normotensive pregnancies [32].

One new and important finding of the present study was that MMP-3 levels were significantly higher in the earlyPre group than in the preeclamptic patients with late-onset preeclampsia (latePre group) and the controls.

MMP-3 is also referred to as stromelysin-1. It digests extracellular matrix components, can activate the latent forms of matrix metalloproteinases, and may induce apoptosis [33]. MMP-3 plays an important role in physiological process of tissue remodelling, but its inappropriate expression can lead to progression of cancer disease [34] and facilitate invasion and metastasis [35] formation.

Mechanisms responsible for controlling trophoblast cells mobility are considered to stimulate production and secretion of MMP-3. On the other hand, inappropriate activation of MMP-3 in the presence of cytokines Th1 such as Il-1b is likely to influence the development of pregnancy complications.

Husslein et al. put forward that active MMP-3 is produced in villous and extravillous trophoblasts of early pregnancy and decreased expression of MMP-3 was observed in later gestation [13].

On the other hand, Reister et al. [3] found strong expression of MMP-3 in trophoblast cells with increased expression in endovascular trophoblast cells in healthy normotensive pregnancies.

It was observed that MMP-3 contributes to trophoblast motility and plays an important role in physiological remodelling processes [13] and trophoblast invasion [12–14]. In contrast reduced MMP-3 expression in the extravillous trophoblast around spiral arteries was observed in pregnancies complicated by severe preeclampsia [3] and in patients with pregnancies complicated by early-onset preeclampsia with IUGR [3].

The aforementioned data from literature and the findings of other researchers along with the results of the present study seem to point to the importance of higher levels of MMP-3 in the development of early-onset preeclampsia, but not in late-onset form of preeclampsia.

It is also feasible that higher maternal levels of MMP-3 in early-onset preeclampsia may cause the disease or ability of compensation for occurring disturbances.

Elevated concentrations of MMP-3 in pregnancies complicated by early-onset preeclampsia and nonaltered ones in pregnancies complicated by late-onset preeclampsia can also indicate the importance of the process of altered trophoblast invasion for the early subtype of the disorder, sometimes called the fetal type, and a less significant role of MMP-3 in late-onset preeclampsia, called well the maternal type, where clinical symptoms appear later. Therefore, it may suggest lower significance of this metalloproteinase in maternal cardiovascular changes and higher significance for placental vessels, in maternal-placental-fetal unit.

Higher levels of MMP-2 and MMP-13 and lower levels of MMP-9 seem to be related to both early- and late-onset preeclampsia.

It seems that proper regulation of metalloproteinases activity is essential to maintain homeostasis of extracellular matrix, and the imbalance of expression and activity of MMPs leads to pathological changes and disorders.

## 5. Conclusions

In summary, the findings of the present study suggest the importance of metalloproteinases in aetiopathogenesis of both early- and late-onset severe preeclampsia. Early- and late-onset severe preeclampsia seem to be associated with increased maternal serum levels of MMP-2 and MMP-13 and decreased levels of MMP-9.

It seems that lower maternal serum MMP-9 levels may lead to abnormal development of blood vessels at the interface between mother and fetus in preeclamptic pregnancies.

Increased MMP-2 levels may reflect ischemic disturbances and vascular endothelial dysfunction in pregnancies complicated by severe preeclampsia or could constitute an attempt to compensate alterations observed in preeclamptic pregnancies.

Furthermore, MMP-3 appears to be involved solely in early-onset preeclampsia. To the best of my knowledge, this is the first study to detect higher maternal serum levels of MMP-3 in patients with early-onset preeclampsia and unaltered levels of MMP-3 in patients with late-onset preeclampsia. These results can also indicate the importance of altered trophoblast invasion only for the early subtype of the disorder.

The potential limitation of the present study is mainly related to the small sample size and the fact that only levels of MMPs were evaluated. The enzymatic activity of the MMPs would be justified to be assessed in order to confirm that higher levels of metalloproteinases are linked to their higher enzymatic activity. The precise determination of a potential role of MMPs in preeclampsia requires further investigation with the application of a larger group of patients.

## Figures and Tables

**Table 1:** The clinical characteristics of early-onset preeclamptic (earlyPre) and late-onset preeclamptic (latePre) patients and control subjects.

	Early-onset preeclamptic patients *[earlyPre]* (*n* = 29)	Late-onset preeclampsia *[latePre]* (*n* = 31)	Healthy controls (*n* = 65) *[control group]*	*p* value
Age (years)	30.66 ± 5.76	28.89 ± 4.61	29.45 ± 4.02	*p* = 0.1811397
Gravidity	2.13 ± 1.73	1.52 ± 0.89	1.46 ± 0.68	*p* = 0.003781^*∗*^
Parity	1.87 ± 1.61	1.40 ± 0.76	1.38 ± 0.56	*p* = 0.020489
Height (cm)	164.26 ± 5.64	164.29 ± 6.22	165.04 ± 5.72	*p* = 0.774341
Maternal weight (kg)	83.64 ± 15.43	82.92 ± 14.50	76.38 ± 13.16	*p* = 0.0374227^*∗*^
BMI (kg/m^2^)	30.78 ± 4.29	30.62 ± 4.65	28.05 ± 4.41	*p* = 0.011356^*∗*^
Systolic blood pressure (mmHg)	165.37 ± 15.28	166.50 ± 18.41	112.51 ± 10.32	*p* < 0.00001^*∗*^
Diastolic blood pressure (mmHg)	109.52 ± 9.75	108.72 ± 9.20	71.92 ± 7.33	*p* < 0.00001^*∗*^
Mean arterial blood pressure, MAPII (mmHg)	128.12 ± 10.53	127.97 ± 11.09	85.09 ± 8.31	*p* < 0.00001^*∗*^
Gestational age at birth (weeks)	30.31 ± 2.41	37.30 ± 1.91	38.18 ± 1.41	*p* < 0.00001^*∗*^
Birth weight (g)	1262.42 ± 437.89	2654.50 ± 713.31	3041.07 ± 527.07	*p* < 0.00001^*∗*^
MMP-2 (ng/mL)	242.93 ± 68.64 Median 235.26 (136.88–466.90)	234.39 ± 79.18 Median 219.17 (124.19–514.79)	195.30 ± 43.53 Median 189.95 (103.88–347.83)	*p* < 0.001^*∗*^
MMP-3 (ng/mL)	63.54 ± 71.58 Median 37.115 (13.65–314.14)	27.91 ± 24.99 Median 18.04 (4.16–124.17)	29.31 ± 58.79 Median 16.749 (2.046–454.53)	*p* < 0.02^*∗*^
MMP-9 (ng/mL)	1450.12 ± 838.01 Median 1153.32 (243.68–3383.13)	1450.86 ± 1165.903 Median 1040.2 (325.07–6430.0)	1752.53 ± 901.54 Median 1605.75 (336.83–4569.2)	*p* = 0.127583
MMP-13 (ng/mL)	0.590 ± 0.681 Median 0.407 (0.054–4.351)	0.482 ± 0.278 Median 0.385 (0.008–1,264)	0.427 ± 0.228 Median 0.409 (0.014–1.047)	*p* = 0.175113

Values are reported as mean ± standard deviation (SD) or median and minimum–maximum; MMP-2: matrix metalloproteinase-2; MMP-3: matrix metalloproteinase-3; MMP-9: matrix metalloproteinase-9; MMP-13: matrix metalloproteinase-13; BMI body mass index (calculated as weight in kilograms divided by the square of height in meters) kg/m^2^. ^*∗*^Statistical significance (*p* < 0.05); groups of studied pregnant women: *control group*: healthy normotensive pregnant women; *earlyPre group*: patients with pregnancy complicated by early-onset severe preeclampsia; *latePre group*: women with pregnancy complicated by late-onset severe preeclampsia.

**Table 2:** Matrix metalloproteinases: MMP-2, MMP-3, MMP-9, and MMP-13 levels in maternal serum samples in both preeclamptic groups of women and in healthy controls.

	MMP-2 (ng/mL)	MMP-3 (ng/mL)	MMP-9 (ng/mL)	MMP-13 (ng/mL)
The *control group* (*n* = 65)	195.30 ± 43.53 Median 189.95 (103.88–347.83)	29.23 ± 58.79 Median 16.749 (2.046–454.53)	1752.59 ± 901.54 Median 1605.75 (336.83–4569.2)	0.427 ± 0.228 Median 0.409 (0.014–1.047)
Statistical analysis, *control-earlyPre*	**p** = 0.000055^**∗**^	**p** = 0.024386^**∗**^	*p* = 0.063965	*p* = 0.093083
The group *earlyPre* (*n* = 29)	242.93 ± 68.64 Median 235.26 (136.88–466.90)	63.54 ± 71.58 Median 37.115 (13.65–314.14)	1450.12 ± 838.01 Median 1153.32 (243.68–3383.13)	0.590 ± 0.681 Median 0.407 (0.054–4.351)
Statistical analysis, *earlyPre-latePre*	*p* = 0.611400	**p** = 0.011567^**∗**^	*p* = 0.997154	*p* = 0.364301
The group *latePre* (*n* = 31)	234.39 ± 79.18 Median 219.17 (124.19–514.79)	27.91 ± 24.99 Median 18.04 (4.16–124.17)	1450.86 ± 1165.93 Median 1040.2 (325.07–6430.0)	0.482 ± 0.278 Median 0.385 (0.008–1,264)
Statistical analysis, *control-latePre*	**p** = 0.002550^**∗**^	*p* = 0.995279	*p* = 0.103238	*p* = 0.295140

Data presented as a mean ± SD. ^*∗*^Statistical significance (*p* < 0.05); groups of studied pregnant women: *control group*: healthy normotensive pregnant women; *earlyPre group*: patients with pregnancy complicated by early-onset severe preeclampsia; *latePre group*: women with pregnancy complicated by late-onset severe preeclampsia.
